# Improvements of Microcontact Printing for Micropatterned Cell Growth by Contrast Enhancement

**DOI:** 10.3390/mi10100659

**Published:** 2019-09-30

**Authors:** Timm J. J. Hondrich, Oliver Deußen, Caroline Grannemann, Dominik Brinkmann, Andreas Offenhäusser

**Affiliations:** 1 Institute of Complex Systems, Bioelectronics (ICS-8), Forschungszentrum Jülich, 52428 Jülich, Germany; t.hondrich@fz-juelich.de (T.J.J.H.); oliver.deussen@rwth-aachen.de (O.D.); caroline.grannemann@rwth-aachen.de (C.G.); do.brinkmann@fz-juelich.de (D.B.); 2 Faculty of Mathematics, Computer Science and Natural Sciences, RWTH Aachen University, 52076 Aachen, Germany

**Keywords:** microcontact printing, patterned neuronal networks, epoxysilane modification, tempering, Glymo, (3-Glycidyloxypropyl)trimethoxysilane (GPTMS)

## Abstract

Patterned neuronal cell cultures are important tools for investigating neuronal signal integration, network function, and cell–substrate interactions. Because of the variable nature of neuronal cells, the widely used coating method of microcontact printing is in constant need of improvements and adaptations depending on the pattern, cell type, and coating solutions available for a certain experimental system. In this work, we report on three approaches to modify microcontact printing on borosilicate glass surfaces, which we evaluate with contact angle measurements and by determining the quality of patterned neuronal growth. Although background toxification with manganese salt does not result in the desired pattern enhancement, a simple heat treatment of the glass substrates leads to improved background hydrophobicity and therefore neuronal patterning. Thirdly, we extended a microcontact printing process based on covalently linking the glass surface and the coating molecule via an epoxysilane. This extension is an additional hydrophobization step with dodecylamine. We demonstrate that shelf life of the silanized glass is at least 22 weeks, leading to consistently reliable neuronal patterning by microcontact printing. Thus, we compared three practical additions to microcontact printing, two of which can easily be implemented into a workflow for the investigation of patterned neuronal networks.

## 1. Introduction

Neuronal cell culture systems have been used in a wide variety of investigations, ranging from single protein studies to the analysis of basic network function. In certain applications, it is beneficial to grow neurons not randomly but in defined patterns. These applications include the exact positioning of neurons on top of the electrodes of microelectrode arrays (MEAs) [1,2,3], the construction of neuronal logical elements [4,5], the investigation of neuronal networks [6,7,8], or the multiplexing of drug tests [9], amongst others.

Neuronal patterning techniques can be divided into different categories, depending on their working principle. On the one hand, the neurons can be spatially confined in microfluidics [10,11,12,13,14] or microchambers [8,15]. In this case, axonal bundles can be monitored very reliably, but it is hard to control neuronal density within the larger chambers, leading to clumping and cluster formation of the neurons. Moreover, seeding the neurons into these chambers can be complicated and time consuming. On the other hand, neuronal growth can be guided by creating cell-attractive and cell-repellent areas either chemically or topographically. Topographies usually influence the outgrowth of neurites but not the positioning of somata, and complex patterns are hard to achieve [15,16,17,18,19]. By tuning the surface chemistry, substrates have been coated with poly-L-lysine (PLL) [1,2,4,20], fibronectine [5,21], laminin [4,5], or other (mostly hydrophilic) molecules [15,19,22,23,24] as cell-attractive agents. As cell-repellent substances, polyvinyl alcohol (PVA) [20], polyethylene glycol (PEG) [21,24], (3-glycidyloxypropyl)trimethoxysilane (Glymo) [1,25], and other (mostly hydrophobic) molecules [5,15,19,23,24,26,27] have been used. These agents have been applied to different substrates such as gold [5,21], glass [1,5,19], or different MEA passivation materials [1,2,19,27,28]. A multitude of different coating procedures have been implemented, depending on the coating, its deposition (sometimes involving additional adhesion layers), and the number or scale of substrates [1,19,20]. Many of these coating procedures rely on photolithographic methods, and are therefore limited to the scale of 4-inch wafers [19,20]. Another widely used way of transferring cell-attractive molecules to a substrate is via microcontact printing (µCP), a comparatively simple method involving fewer photolithographic steps.

Stamps for µCP are fabricated by casting a polymer into a mold that is usually prepared using photolithographic techniques [4,19,21,27]. This procedure can be repeated multiple times with the same mold, increasing the scale of the actual stamp production above the scale of 5-inch wafers. After this, the stamps are bathed in a solution of coating molecules, which are then transferred to a substrate. In 1997, µCP was used to transfer cell-attractive molecules to a cell-repellent surface [21]. Later, neuronal cultures were also patterned with spatial resolutions, ranging from individual dendrites [24,26,29,30,31,32,33] or even dendritic spines [34] to whole populations [4,5]. However, the reliability and effectiveness of µCP-based patterning highly depends on the substrate, the molecules used, and the pattern. To improve the reliability of the patterning in our applications, we aim to enhance the contrast between the cell-attractive µCP pattern and the cell-repellent background. This enhancement should rely on simple procedures that can be integrated into standard cell culture work, increasing independence from nanotechnological and cleanroom techniques.

We tested three different, easily implementable additions to standard µCP processes on borosilicate glass, two of which enhance the µCP quality significantly in our system. As a first approach, we tested the precipitation of manganese salt for background toxification but found that this technique does not allow for any patterning neurons. The second approach is based on the heat-induced inactivation of the substrate which successfully hydrophobized the substrate and allowed for improved patterning. To chemically uncouple the glass surface from the cells and coating, we based the third approach on the covalent binding of epoxy silanes to the activated glass surface as published by Nam et al. [1], and the subsequent functionalization of these silanes with hydrophilic, cell-attractive poly-L-lysin (PLL). As a novel addition, we additionally coupled the remaining epoxy silanes in the background of the patterns to hydrophobic, cell-repellent dodecylamine (DDA). We reproduce the enhanced quality of the patterns and demonstrate the capability of this method for long-term storage of many silanized glass substrates, making it a scalable, easy addition to standard µCP.

## 2. Materials and Methods

### 2.1. Stamp Fabrication

The dark-field chrome mask for different patterns was produced via electron beam writing. A dehydrated 0.6-mm-thick standard silicon wafer (5″ diameter, MEMC Electronic Materials, O’Fallon, MO, USA) was spin coated with 5 to 12 µm of AZ4562 photoresist (Clarion GmbH, Mörfelden-Walldorf, Germany), and dried for 60 s at 130 °C. The mold for stamp production was fabricated by transferring the patterns from the chrome mask to the coated wafer using UV photolithography. The wafer was baked for 90 s at 140 °C. The development of the resists was conducted in MF-24-A (Süss MicroTec, Garching near Munich, Germany) for 50 s and stopped by a washing step in Milli-Q water. To complete the mold, reactive ion etching with SF_6_ at 150 W for 10 min was used to transfer the patterns into a depth of 4.5 µm. Finally, a release layer of (tridecafluoro-1,1,2,2-tetrahydrooctyl)trichlorosilane (FOTCS) (Sigma Aldrich, Munich, Germany) was deposited on the surface of the mold using chemical vapor deposition at room temperature, and 45 mbar for 1.5 h. polyolefin plastomer (POP) stamps were produced using hot embossing, as described previously [4,34].

### 2.2. Chemical Vapor Deposition (CVD) of Silanes

CVD was conducted according to [35]. In brief, glass coverslips (VWR, Radnor, PA, USA) were cleaned in 2% Hellmanex detergent (Hellma GmbH & Co. KG, Müllheim, Germany) in an ultrasound bath, rinsed three times with Milli-Q water, and washed once with Milli-Q water in an ultrasound bath. After transfer of the coverslips to ethanol (EtOH), they were dried with nitrogen and activated in a plasma oven for 30 min at 0.7 mbar and 80 W power. The activated coverslips were transferred to a desiccator with a pre-heated ceramics ground disc and glass beaker (200 °C) in a water- and oxygen-free argon atmosphere. To the desiccator, 150 µL of Glymo (Sigma Aldrich, Munich, Germany) were added, and the deposition was conducted for 1.5 h at 5 mbar.

### 2.3. Heat Treatment

Glass coverslips (VWR, Radnor, PA, USA) were placed in a sample holder and baked at 300 °C for 5 h. The coverslips were passively cooled overnight in the oven to avoid breakage due to thermal stress.

### 2.4. Microcontact Printing

The POP stamps were cleaned with 70% EtOH for 10–15 min in an ultrasound bath and washed with Milli-Q water. The stamps were quickly dried with nitrogen and incubated on ice for >20 min in an appropriate amount of Hank’s balanced salt solution (HBSS) containing 10 µg/mL PLL labeled with the green fluorescent dye fluorescein isothiocyanate (FITC; Sigma Aldrich, Munich, Germany). Excess liquid on the patterned side of the stamps was dried with nitrogen, and the stamps were placed on the modified or untreated glass substrate. A weight of 40 g was placed on the stamps for 2 min to ensure adhesion, and the stamps were incubated for >20 min on the substrate. For Glymo-treated samples, the samples were either bathed afterwards for >20 min in DDA and washed three times with EtOH or just washed three times with EtOH.

Before placing the printed substrates into a cell-culture plate, the well of the plate was coated homogeneously with 10 µg/mL unlabeled PLL in Hank’s balanced salt solution (HBSS; Sigma Aldrich, Munich, Germany) for 20–30 min at room temperature. It was washed three times with HBSS, and the liquid was completely aspirated. This ensures a neuronal co-culture outside of the actual pattern and a more beneficial environment for the cells.

### 2.5. Capillary Deposition of MnO_2_

After placing the POP stamp on the glass substrate during µCP, the spaces between patterns were back-filled with a 0.1 w%, 1 w%, 3 w%, or 5 w% KMnO_4_ solution. For this, a drop of the solution was placed onto the substrate at the edge of the stamp and allowed to penetrate into the space between the µCP pattern via capillary forces. The solution under the stamps was dried to allow for precipitation and decomposition to MnO_2_ at room temperature overnight.

### 2.6. Contact Angle Measurements

Contact angles of surfaces were measured with a Contact Angle System OCA (dataphysics, Filderstadt, Germany) or with a Lumix camera (Panasonic, Kadoma, Japan) on a custom-built setup. Image analysis and angles were determined semi-automatically with the SCA Software or manually with ImageJ [36].

### 2.7. Light Microscopy

Fluorescence microscopy was used to evaluate the transfer of PLL-FITC onto a substrate. An Axio Imager Z1 (ZEISS, Oberkochen, Germany) was used in combination with an HXP metal halide light source and appropriate optical filters for visualizing FITC.

Phase contrast microscopy was conducted at an Axiovert 200 (ZEISS, Oberkochen, Germany) to monitor cell growth inside and outside of the patterns.

### 2.8. Cell Culture

Isolation of primary cortical neurons from E18 Wistar rats has been described elsewhere [37]. The isolated cells were transferred to Neurobasal medium (Life Technologies, Carlsbad, CA, USA) supplemented with 1% (*v*/*v*) B27 (Thermo Fisher Scientific (Gibco), Waltham, MA, USA), 0.5 mM L-glutamine (Thermo Fisher Scientific (Gibco), Waltham, MA, USA) and 0.05 mg/mL Gentamicin (Sigma Aldrich, Munich, Germany), and seeded with a density of roughly 150 cells/mm² onto the microcontact printed substrates. The cultures were incubated at 37 °C and 5% CO_2_ for up to one day in vitro (DIV) 21. The complete Neurobasal medium was exchanged ~2 h after seeding, and 50% of the medium was again exchanged twice per week.

### 2.9. Live-Dead Stainings

Cultured cells (DIV 4) were incubated at 37 °C in 1 μg/mL Calcein acetoxymethyl ester (Calcein-AM) and 2 μM ethidium homodimer (EtHD) (both Life Technologies, Carlsbad, CA, USA) in Neurobasal medium for 15 min. The medium was exchanged once before imaging with an Axio Imager Z1 (ZEISS, Oberkochen, Germany) fluorescence microscope. Micrographs were analyzed with the Fiji (Image J) (version 1.52n, open source software, maintained by Laboratory for Optical and Computational Instrumentation, University of Wisconsin-Madison, Madison, WI) [36] plugin “Analyse Particles” after binarization of the image with the “Thresholding” plugin. All images were analyzed using the same parameters.

## 3. Results and Discussion

**Figure 1:**
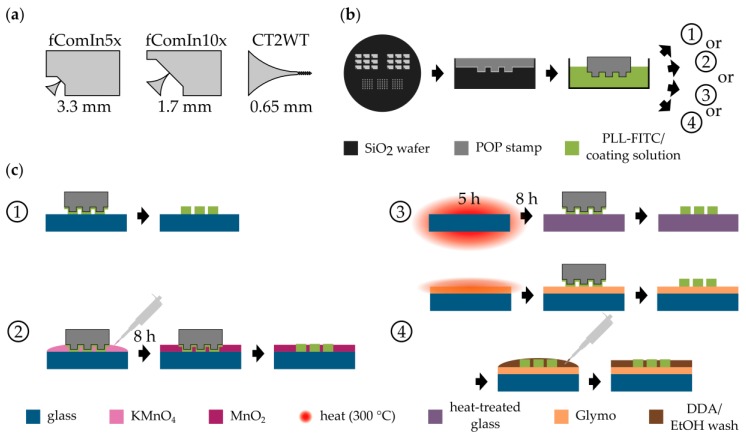
Modified µCP processes. (**a**) Different patterns for µCP used in this study of different sizes. (**b**) Generally applicable steps of µCP. The patterns are etched into a silicon wafer and the polymer is cast into the wafer. The hardened polymer is cut into stamps and the stamps are incubated in a coating solution. (**c**) Variants of µCP. Variant 1 is the standard process. The coating solution is transferred to an unmodified glass coverslip. In variant 2, the stamp is placed on the substrate and a KMnO_4_ solution is applied to the substrate. After precipitation overnight, the stamp is removed, leaving the pattern surrounded by a layer of water insoluble MnO_2_. In variant 3, the glass coverslip is heated for 5 h and cooled overnight. The modified substrate is stamped like in variant 1. Here, the incubation step in (**b**) takes place just before the printing step. In variant 4, a Glymo monolayer is applied to the coverslip via CVD (chemical vapor deposition) and the coating is transferred to the Glymo layer. The substrate is either washed with EtOH or incubated in dodecylamine (DDA) and afterwards washed with EtOH so that unoccupied Glymo is bound.

**Figure 2:**
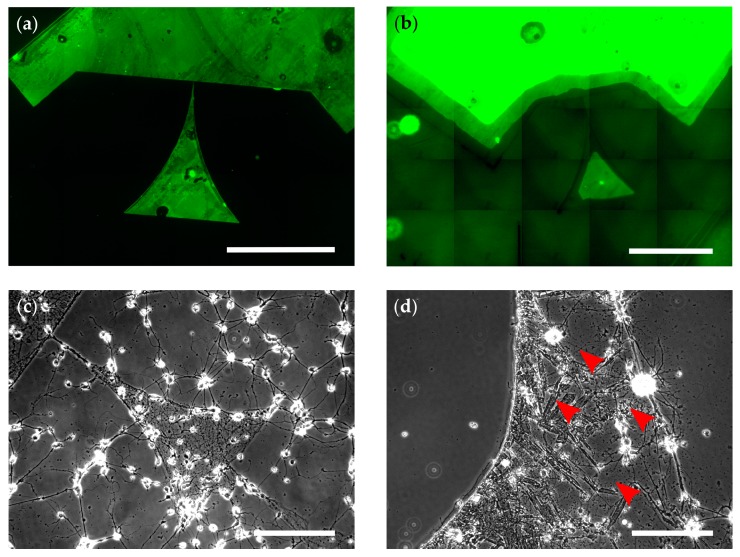
Patterning on untreated glass and with deposition of MnO_2_ (compare Figure 1c). (**a**,**b**) Fluorescence micrographs of microcontact printed, patterned PLL-FITC (poly-L-lysin-fluorescein isothiocyanate) (green) on untreated glass (**a**; scale bar: 400 µm) or glass with a background of MnO_2_ (**b**; scale bar 500 µm). (**c**,**d**) Phase contrast micrographs of neurons growing within patterns on untreated glass (**c**; DIV 6) or glass with a background of MnO_2_ (**d**; DIV 7). Scale bars: 100 µm. (**d**) Red arrowheads indicate abnormal neurites.

For the generation of patterned cell cultures, µCP is a well-established technique [19]. As seen in Figure 1a, the patterns used in this study are based on triangular patterns previously published by us, which enable directional signal propagation of neuronal populations [4]. During µCP, a cell-attractive coating (in our case, PLL-FITC) is transferred with a polymer stamp to a substrate that is inherently cell-repellent or can be made cell-repellent, as seen in Figure 1b,c variant 1. In this study, POP is used as the stamp polymer because its increased stiffness, when compared with polydimethylsiloxane (PDMS), makes it less likely to touch the substrate at places outside of the µCP patterns. The technique of µCP relies on the cell-attractive properties of the pattern and the cell-repellency of the background [23]. Therefore, we tested different novel approaches to improve the cell-repellency of the background and stabilize the patterned coating when using µCP. Two of these approaches are promising, easy-to-implement additions to the standard procedure of creating patterned neuronal networks. As a basis and control for these improvements, we chose commonly used borosilicate glass because it serves as a substrate, for example, in imaging techniques [4,5,6] applied to micropatterned cell cultures. Moreover, due to its component SiO_2_, it serves as a model system for SiO_2_-based surface materials of sensors such as microelectrode arrays [1]. Because of the variable production process of borosilicate glass coverslips, the patterning is not reliably reproducible on this substrate. Although µCP patterns lead to well-grown cultures on some batches of coverslips, in others, cells also attach to the background, as seen in Figure 2c.

### 3.1. Background Toxification via Water-Insoluble MnO_2_

Bulk metals, such as molybdenum, silicon as well as different metal nanoparticles, show cytotoxic effects in cell culture systems [38,39]. On the basis of these facts, we decided, as a first unorthodox approach, to toxify the background surface of the µCP pattern. This was predicted to lead to an increased contrast between healthy cells in the pattern and dead cells on the toxified background. Such a toxification agent has to fulfill certain criteria: (1) It has to be depositable; (2) It has to be water-insoluble in the deposited state; (3) It has to be neurotoxic. To match these criteria, we chose to use a manganese salt system. Potassium permanganate (KMnO_4_) is soluble in water and can precipitate (i.e., deposit) onto a surface via a decomposition reaction:

(1) $$2\;{\mathrm{KMnO}}_{4}+H_{2}O\to 2\;{\mathrm{MnO}}_{2}+1.5\;O_{2\left(g\right)}+2\;\mathrm{KOH}$$

The resulting salt MnO_2_ was water insoluble and neurotoxic [39,40]. Therefore, the system satisfies all three conditions.

To implement this salt system into the µCP process, we distributed KMnO_4_ in the background of the pattern using the capillary forces of the POP stamp positioned on the glass substrate, as seen in Figure 1b,c, variant 2. After precipitation to MnO_4_ overnight, the stamp was removed, the transfer of the pattern was checked, and neurons were seeded onto the substrate.

Fluorescence microscopy showed that the patterns were successfully transferred to the substrate, as seen in Figure 2b. However, the pattern edges were degraded or missing when compared with the standard process, as seen in Figure 2a. This degradation could, for example, result from an oxidation process during precipitation because KMnO_4_ is a strong oxidation agent. Further, we investigated cell growth at DIV 0, 1, 2, 7, 14, and 21. The cells did not grow within the pattern and showed abnormal neurite morphologies (grainy or thick and unusually straight), as seen in Figure 2d (red arrowheads) when compared with the control seen in Figure 2c. The few growing cells were located in the background areas with precipitated MnO_4_. Thus, the toxic effect of MnO_2_ is not strong enough as an insoluble surface coating, possibly due to a lack in cellular uptake of diffusible ions [41]. The cells might adhere better to the MnO_4_ substrate because the salt crystals roughen the surface or create a surface topography [18,42].

### 3.2. Heat-Induced Background Hydrophobization of Glass

**Figure 3:**
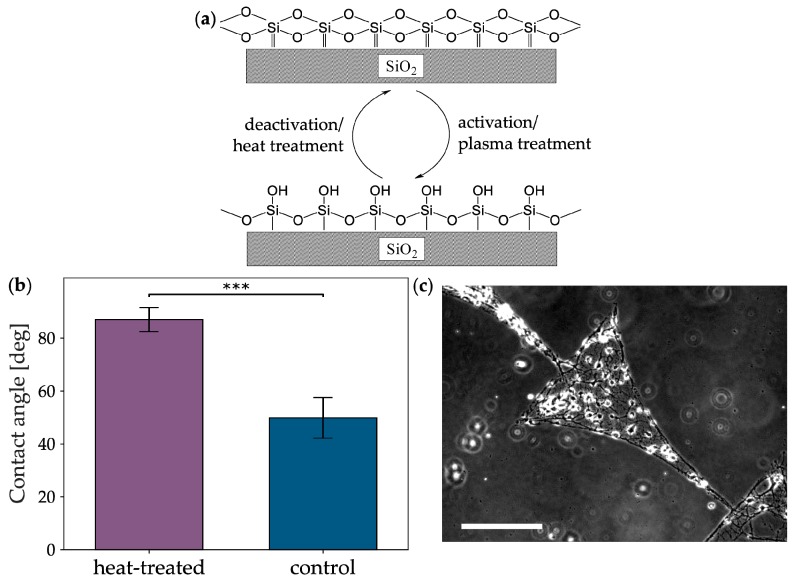
Theory and effects of heat-treating glass for patterned neuronal cultures. (**a**) Activation and deactivation processes on an idealized glass surface comprising only silicon oxide. By providing the activation energy of a condensation reaction between the surface hydroxyl groups with heat treatment, the glass surface should be deactivated and become less hydrophilic. (**b**) Difference of contact angle between heat-treated and untreated glass (control). Color code of the bars corresponds to Figure 1. Statistical significance was tested with the Mann-Whitney U test (p = 9.1 × 10^−5^; n = 10). (**c**) Phase contrast micrograph of neurons growing within pattern on heat-treated glass at DIV6. Scale bar: 100 µm.

After the unsuccessful patterning with MnO_2_, we switched to the more classical approach of changing the surface chemistry of the glass itself. Borosilicate glass is the most common material for laboratory materials, such as microscopy coverslips. Because its intended use is mostly in macroscopic applications, its surface chemistry is disregarded during commercial production. Thus, the production may vary slightly between batches. One step that can vary quite easily is a heat treatment or tempering step. If the temperature during or the cooling after this step varies, the glass becomes more or less hydrophilic due to the amount of free hydroxyl groups on the surface, as seen in Figure 3a. Because cells usually adhere to moderately hydrophilic surfaces [23], such a hydrophilic surface on the entire glass coverslip would lead to inaccurate patterning, as seen in Figure 2c, whereas a hydrophobic surface would lead to accurate patterning.

To deactivate and thereby hydrophobize the surface, we introduced an additional 5 h heating step at 300 °C, followed by slow cooling overnight into the µCP process, as seen in Figure 1b,c, variant 3. This heat treatment provides the activation energy for a condensation reaction between two hydroxyl groups [43], thereby deactivating the surface, as seen in Figure 3a. To validate the effect of the heat treatment, we determined the hydrophobicity of the glass by measuring the contact angle (θ) of the water-glass interface before and after heat treatment, as seen in Figure 3b. Indeed, the hydrophobicity of heat-treated glass (θ = 87.0° ± 4.6°) was increased significantly (p = 9.1 × 10^−5^, Mann-Whitney U test) when compared with the control (θ = 49.9° ± 7.7°). Moreover, the variability of θ seems to have been slightly reduced in the case of treated glass. This indicates that, even within a single batch of glass, the production-based variability is high, and that this variability can be decreased by equalizing the surfaces of the coverslips via heat to a more hydrophobic level.

To test if the increased hydrophobicity of the glass had the desired effect on neuronal patterning, we cultured neurons on heat-treated substrates and evaluated the culture on DIV 6. A clear distinction between the pattern and the background could be seen, as seen in Figure 3c. Unexpectedly, the ratio between alive cells and the sum of alive and dead cells on bare heat-treated substrates (6.71% ± 5.15%) or heat-treated substrates coated homogeneously with PLL (4.17% ± 3.76%) was not different, thereby not reflecting the enhanced quality of patterning, as seen in Supplementary Figure S1. This contradiction probably arises from the fact that heat treatment still heavily relies on the initial surface properties of the glass and may vary between glass batches. Moreover, the process of µCP might attach the PLL more effectively to the surface than a bath application. Nevertheless, the improved patterning quality shows the effectiveness of this modification of µCP.

### 3.3. Glymo Silanization for Covalent Bonding of Amino Acids on a Cell-Repellent Background

Although the additional heat treatment during µCP offers an easy way to improve the effectiveness of the technique, it still depends on the fabrication, composition, and surface properties unique to a particular batch of glass. Moreover, the deactivation of the silicon dioxide reverts with prolonged exposure to oxygen. Also, a coating molecule like PLL is still only physisorbed to the surface [24] and does not covalently bound, which could lead to a weak cell adhesion to the substrate. Although the last point is important on glass coverslips, it is essential for µCP on MEAs to provide a good coupling of the cells to the electrodes.

**Figure 4:**
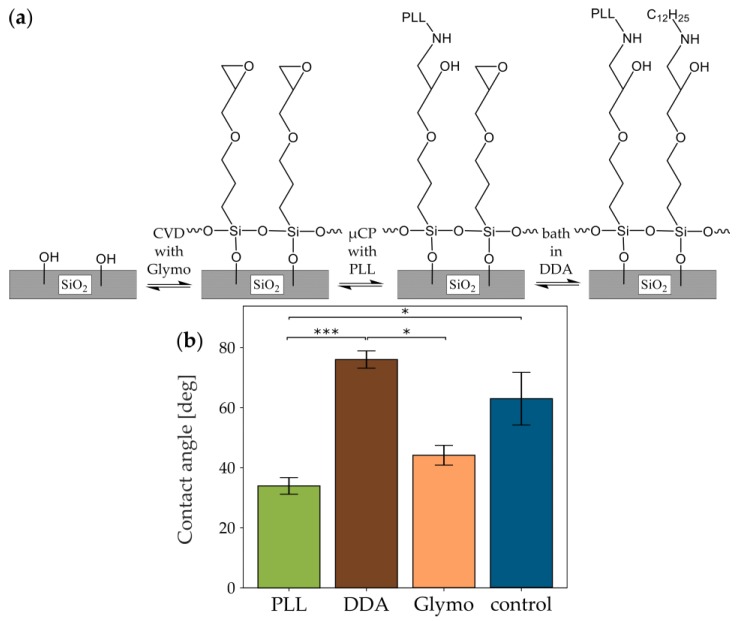
Theory and effect of glass treatment with Glymo and different secondary molecules. (**a**) Reaction schematics of the Glymo-based modification of idealized glass. First, Glymo is applied by CVD followed by the binding of PLL via µCP. Finally, DDA is bound to the unbound epoxy groups of Glymo. Alternatively, Glymo remains unbound in a washing step with EtOH. (**b**) Difference of contact angle between glass with Glymo and a homogeneous secondary treatment with PLL, DDA, or EtOH, and untreated glass (control). Color code of the bars corresponds to Figure 1. Statistical significance was tested for all samples with the Kruskal-Wallis H-test for independent samples (p = 0.0001; n = 6). For individual comparison, Dunn’s multiple comparison test with Bonferroni correction was used (p_PLL/DDA_ = 9.05 × 10^−5^; p_PLL/EtOH_ = 0.9183; p_PLL/control_ = 0.0225; p_DDA/EtOH_ = 0.0225; p_DDA/control_ = 0.9183; p_EtOH/control_ = 0.8499).

To simplify the covalent bonding from a three-step [21] to a two-step procedure, we modified an approach based on the functionalization of the glass with Glymo, as seen in Figure 1b,c, variant 1, first published by Nam et al. [1]. In brief, we activated the glass surface via oxygen plasma and deposited Glymo onto the activated glass. In this step, the silane backbone of the Glymo should bind to the hydroxyl groups of the activated glass, as seen in Figure 4a, in a condensation reaction [44]. Also, the silane groups of the individual Glymo molecules interconnect to form a stable monolayer [45]. Next, PLL was microcontact printed onto the Glymo layer with POP stamps, which allows for a reaction between the amino group of PLL and Glymo’s epoxy group [1]. Finally, the samples were bathed in DDA and washed with EtOH, or directly washed with EtOH. This additional step should lead to the condensation reaction of the amino group of DDA to Glymo’s epoxy group to increase hydrophobicity and prevent the epoxy ring from hydrolysis by EtOH or water in subsequent steps or storage until seeding. On the other hand, direct washing with EtOH should not modify Glymo’s epoxy group.

#### 3.3.1. Glymo-Functionalized Glass Improves the Contrast between Pattern and Background

We tested the success of the functionalization strategy by measuring the glass’s contact angle (θ) of the water-glass interface in four different conditions corresponding to the surfaces in contact with cells (untreated glass/control, Glymo+PLL, Glymo+DDA, Glymo+EtOH). The hydrophobicity of the surfaces increases in the order PLL < EtOH < control < DDA (34.0° ± 2.8° < 44.1° ± 3.3° < 62.9° ± 8.8° < 76.0° ± 2.9°; Figure 4b). The standard deviation of θ on the control is with 8.8° more than twice as large as the other standard deviations. This adds further evidence to the theory that untreated glass has a variable surface even within one batch. The inter-batch variability of untreated glass can be seen from the difference in θ between the control group for Glymo (Figure 4b; θ = 62.9° ± 8.8°) and the control group for heat-treated glass (Figure 3b; θ = 49.9° ± 7.7°). Thus, although θ on the untreated glass of this particular batch is not statistically significantly different from θ on DDA or EtOH, its variability is reduced by applying these molecules. Expectedly, PLL strongly decreases the hydrophobicity, creating a cell-attractive environment. Most importantly, the highest difference in hydrophobicity is present between PLL and DDA functionalizations but not between PLL and EtOH. This indicates that the pattern-background separation should most strongly pronounces in DDA-treated samples.

**Figure 5:**
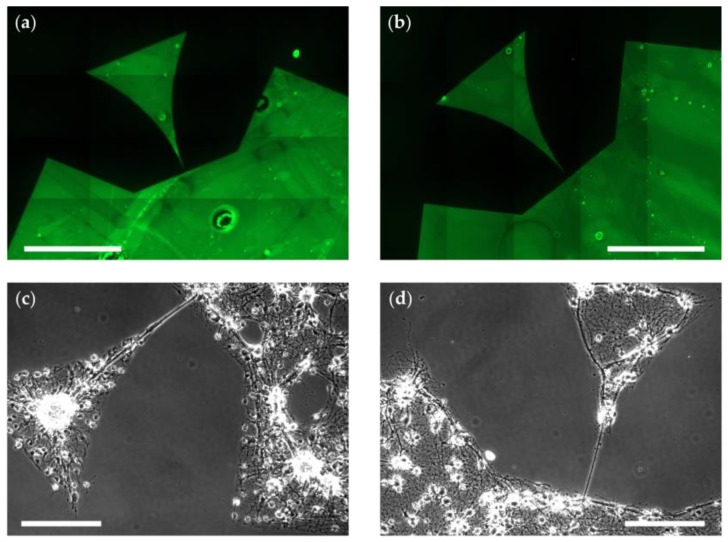
Patterning on glass with deposited Glymo. (**a**,**b**) Fluorescence micrographs of microcontact printed, patterned PLL-FITC (green) on glass with deposited Glymo, bathed in DDA (after µCP), and washed three times with EtOH (**a**) or only washed with EtOH (**b**). Scale bars: 500 µm. (**c**,**d**) Phase contrast micrographs of neurons growing within patterns on glass treated as in (**a**) (**c**; DIV 14) or glass treated as in (**b**) (**d**; DIV 14). Scale bars: 100 µm.

**Figure 6:**
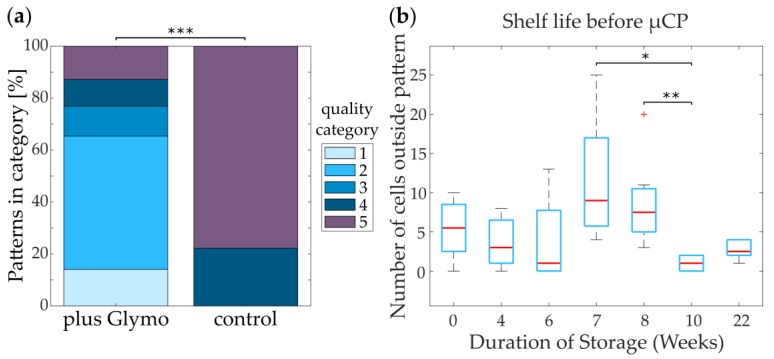
Patterning efficiency and long term stability of neurons on glass functionalized with Glymo and PLL, followed by DDA. (**a**) Amount of cell patterns at DIV 5 in different quality categories after printing and neuronal growth (see Supplementary Figure S4; n = 78) when compared with untreated glass (control; n = 9). Statistical significance was tested with the Mann-Whitney U test (p = 9.6 × 10^−6^). (**b**) Long term shelf life of Glymo-functionalized glass, measured by the number of cells growing outside of patterns at DIV 5 after up to 22 weeks of substrate storage before µCP. Statistical significance was tested for all samples with the Kruskal-Wallis H-test for independent samples (p = 0.0010). For individual comparison, Dunn’s multiple comparison test with Bonferroni correction was used (p values < 0.05: p_week7/week10_ = 0.0044; p_week8/week10_ = 0.0237; see Supplementary Table S1).

To test the influence of the different surfaces on actual cells, we seeded neuronal cultures on substrates treated with only EtOH or additional DDA and checked the cells until DIV 14. Both the pattern itself (Figure 5a,b) and the cells growing in the pattern (Figure 5c,d) showed very clear borders and few cells growing on the background. This clear distinction between pattern and background was present from DIV 0 until DIV 14 (Supplementary Figure S2). Surprisingly, there seemed to be no difference between DDA (Figure 5 a,c) and EtOH (Figure 5 b,d). However, based on the contact angles we decided to use the DDA-treated samples for further analysis, as they might be more stable in different glass batches. Determining the ratio between alive cells and the sum of alive and dead cells on substrates homogeneously coated with Glymo and PLL or Glymo and DDA further verified the cell-attractive and cell-repellent properties of the pattern and the background, respectively. Both the live-dead ratio and the total number of cells was higher on PLL-coated substrates (ratio: 28.93% ± 10.08%; total number: 7259 ± 1703) than on DDA-coated ones (ratio: 14.32% ± 7.83%; total number: 3308 ± 194; Supplementary Figure S1). To quantify the differences in pattern qualities, we divided patterns on DDA and on untreated glass into 5 different quality categories based on the number of cells growing outside the patterns and their attachment to the patterned cells (see Supplementary Figure S3 for details). The five quality categories range from 1 (functional pattern without living cells growing outside of the pattern) to 5 (no pattern visible). Patterned cells on Glymo- and DDA-treated substrates show a significantly higher quality (median = category 2) of the patterns as compared to control (median = category 5; Figure 6a).

#### 3.3.2. Glymo Is Long-Term Stable on Glass

Because the functionalization of glass with Glymo is a time-consuming procedure, we tested whether Glymo could be deposited onto a large amount of coverslips and stored in ambient conditions for later use. The storage of Glymo of up to 22 weeks did not decrease its functionality after µCP in terms of the number of cells growing outside of the pattern, as seen in Figure 6b and Supplementary Table S1, the categorization of quality, as seen in Supplementary Figure S4a, or the number of neurites growing outside of the pattern, as seen in Supplementary Figure S4b and Supplementary Table S2. Samples used at different weeks after Glymo deposition show statistically significant variations in either dendrites outside of the pattern (week 0 versus week 4) or cells outside of the pattern (week 7 versus week 10; week 8 versus week 10). However, since these irregularities vary between the different quantifications and are completely missing from the quality analysis, as seen in Supplementary Figure S4a, they are probably caused by variations in µCP, cell seeding, or neurons themselves. Thus, Glymo can be stored over months, leading to an increased efficiency of this addition to µCP.

## 4. Conclusions

In this study, we tested three different modifications of µCP used for neuronal cell culture patterning. Firstly, MnO_2_ is not suitable for background toxification, and cellular patterning by µCP is not possible with this modification. Secondly, heat treatment of glass coverslips shows an increased hydrophobicity of the substrate, and the pattern quality is improved. Thirdly, application of Glymo as a linker between glass and PLL, accompanied by a subsequent bath in DDA for hydrophobization greatly improves patterning quality. Moreover, these Glymo-modified coverslips have a long shelf life, increasing the efficiency of this method. Thus, these modifications provide valuable tools for simply increasing the effectiveness and efficiency of µCP as a technique for neuronal network investigation.

## Supplementary Materials

The following are available online at https://www.mdpi.com/2072-666X/10/10/659/s1, Figure S1: Analysis of cell survival on different substrates, Figure S2: Additional timepoints in growth on glass with deposited Glymo, Figure S3: Examples of patterns in five different quality categories at DIV 5, Figure S4: Alternative representations of the long-term stability of Glymo on glass, Table S1: Results of Dunn’s test with Bonferroni corrected p values for cells outside of patterns established with Glymo and DDA background hydrophobization, Table S2: Results of Dunn’s test with Bonferroni corrected p values for neurites outside of patterns established with Glymo and DDA background hydrophobization.
