# Supplementary material for: Improvements of Microcontact Printing for Micropatterned Cell Growth by Contrast Enhancement
Source: Micromachines (Basel). 2019 Sep 30;10(10):659. doi: 10.3390/mi10100659 (PMC6848919; doi:10.3390/mi10100659)
Supplement: Supplementary file 1 [file micromachines-10-00659-s001.pdf]

micromachines

Supplementary Materials: Improvements of Microcontact Printing for Micropatterned Cell Growth by Contrast Enhancement

Timm J. J. Hondrich [1,2], Oliver Deußen [1,2], Caroline Grannemann [1,2], Dominik Brinkmann [1,2] and Andreas Offenhäusser [1,*]

1 Institute of Complex Systems, Bioelectronics (ICS-8), Forschungszentrum Jülich, Jülich, Germany
2 Faculty of Mathematics, Computer Science and Natural Sciences, RWTH Aachen University, Aachen, Germany
* Correspondence: a.offenhaeusser@fz-juelich.de; Tel.: +49-246-161-2330

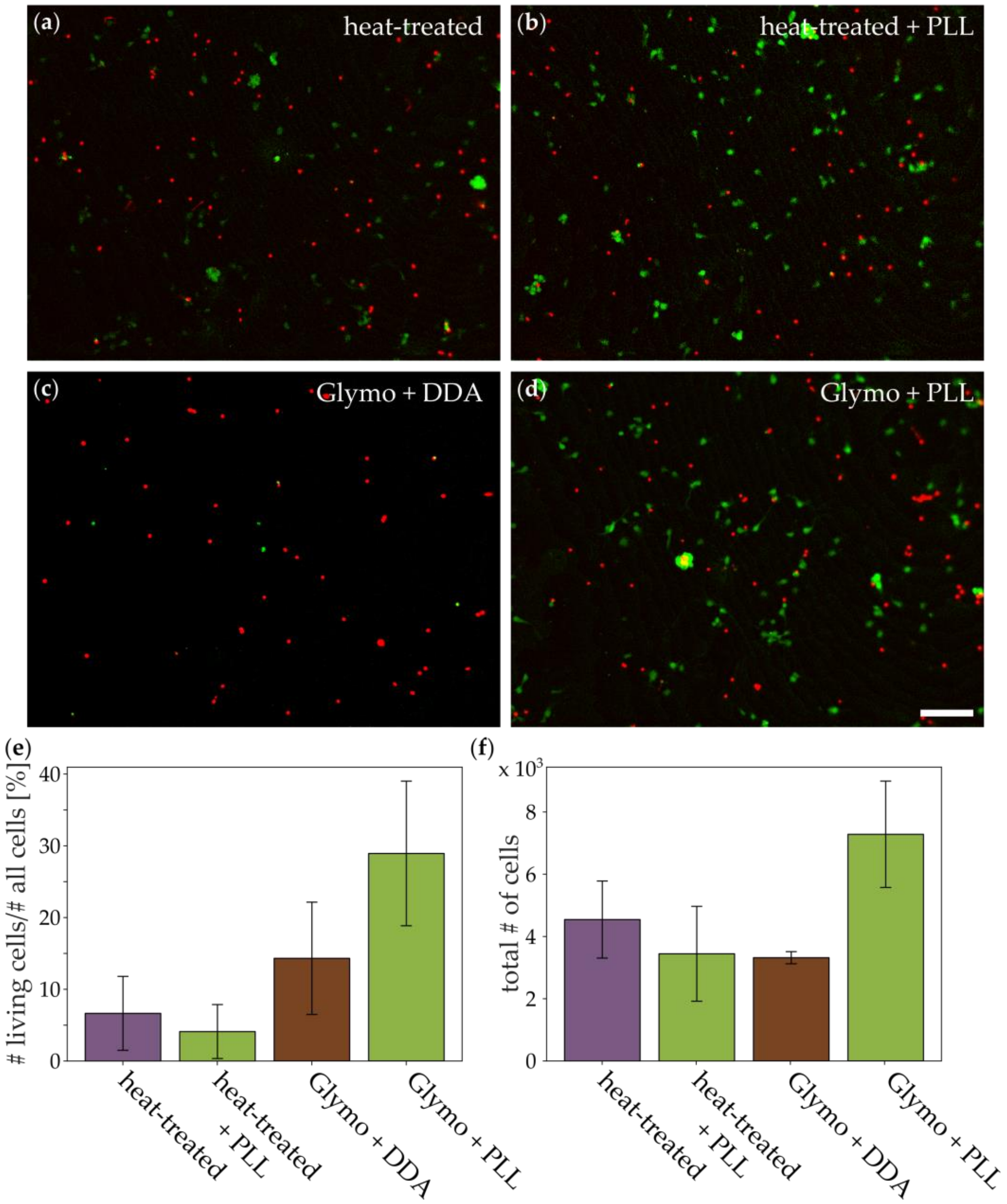

Figure S1. Analysis of cell survival on different substrates. (**a**–**d**) Fluorescent micrographs of neurons growing on heat-treated glass without (a) or with PLL (b), or Glymo-treated glass bathed in DDA (c) or PLL (d). The neurons were stained with Calcein-AM (living cells, green) and EtHD (dead cells, red). Scale bar in (d) applies for (a–d): 100 µm. (**e**) Ratio of the number of living cells and the sum of dead and living cells (N = 3 cell cultures per condition, as depicted in (a–d)). (**f**) Total number of living and dead cells, corresponding to (e).

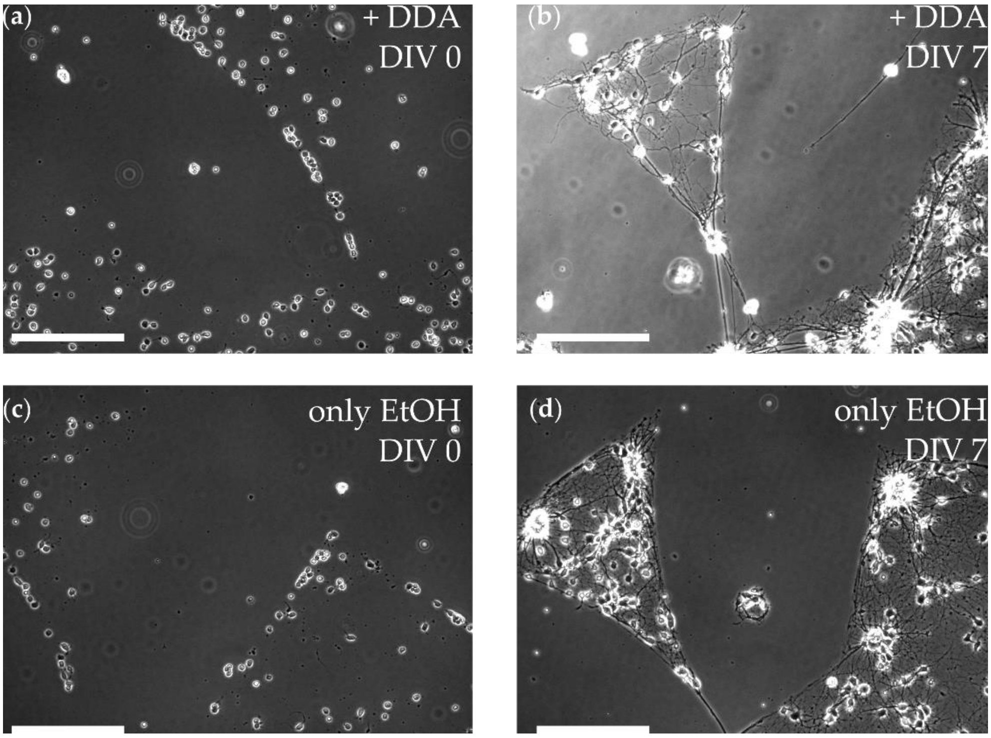

Figure S2. Additional timepoints in growth on glass with deposited Glymo. (**a**–**d**) Phase contrast micrographs of neurons growing within patterns on glass treated with DDA (after μCP), and washed three times with EtOH (a, DIV 0; b, DIV 7) or only washed with EtOH (c, DIV 0; d, DIV 7). Scale bars: 100 μm.

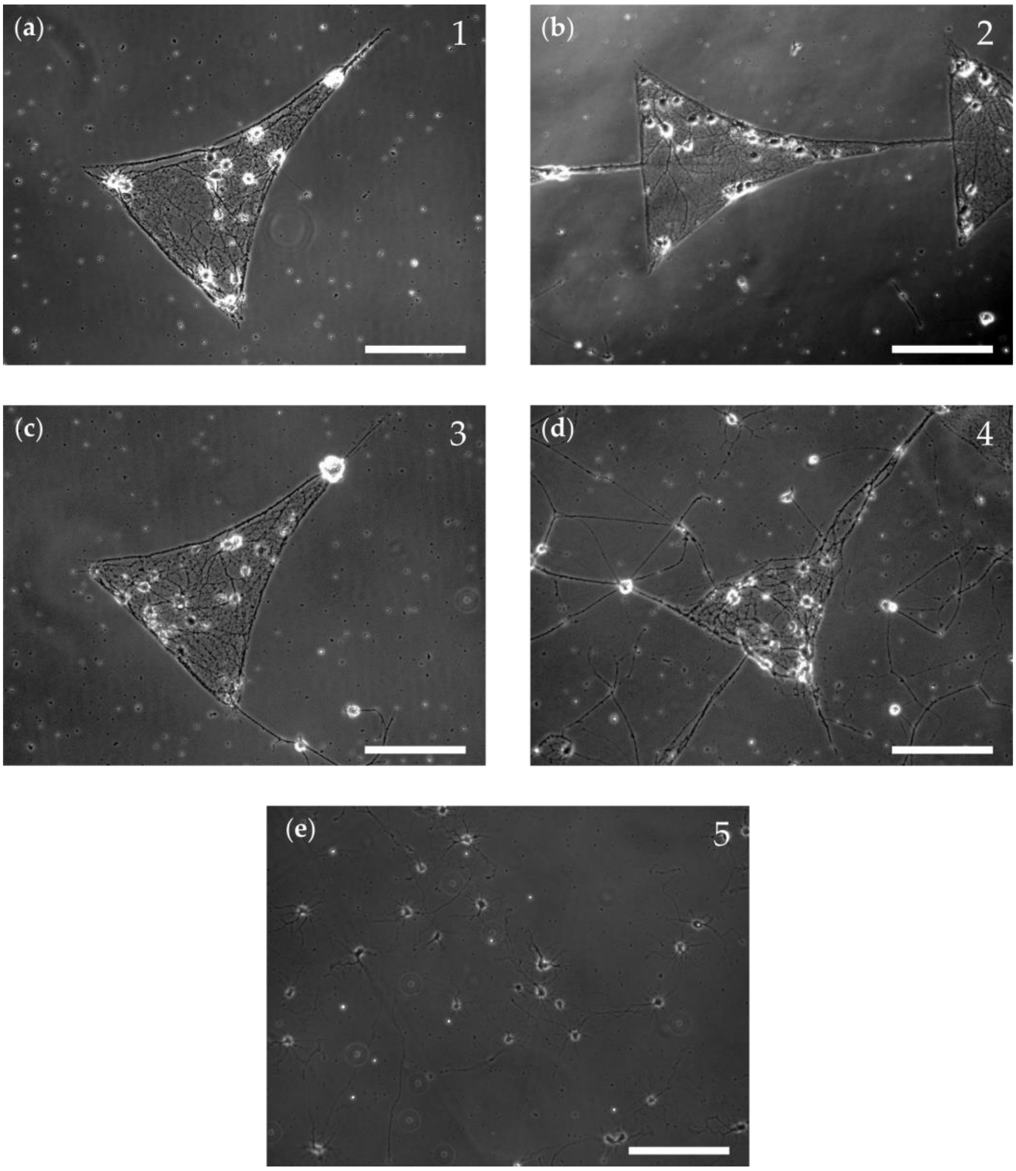

Figure S3. Examples of patterns in five different quality categories at DIV 5. (**a**) 1: Only dead cells are outside of the pattern. (**b**) 2: Less than 5 cells are outside of the pattern in the field-of-view (not connected to the pattern). (**c**) 3: Less than 5 cells are outside of the pattern in the field-of-view (connected to the pattern). (**d**) 4: More than 5 cells are outside of the pattern in the field-of-view but the majority of cells/dendrites was within pattern. (**e**) 5: No pattern was visible, and cells grew homogeneously or were dead. Scale bars: 200 µm.

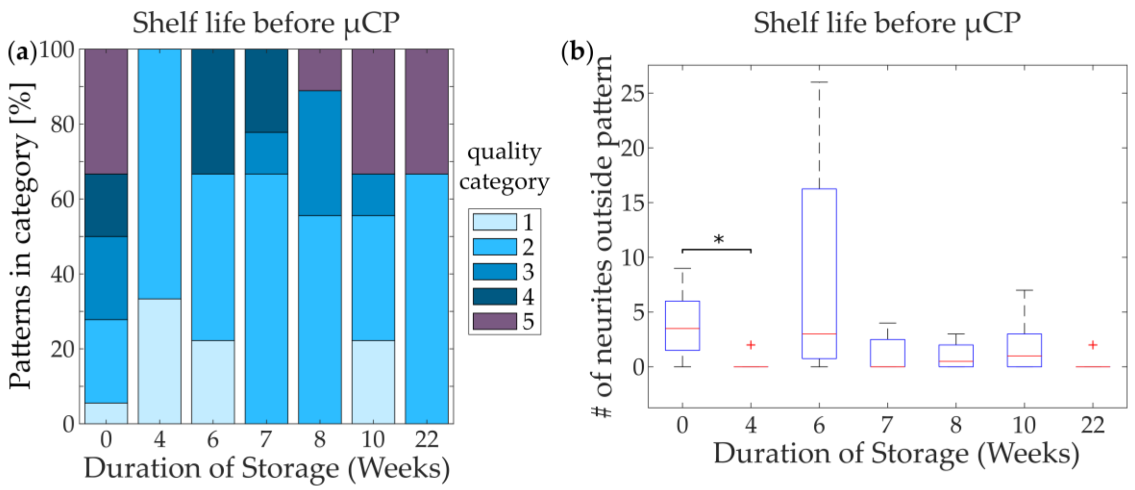

Figure S4. Alternative representations of the long-term stability of Glymo on glass. Glymo-functionalized glass can be used up to at least 22 weeks of storage before μCP. (**a**) Long-term shelf life of Glymo-functionalized glass, measured by the amount of cell patterns at DIV 5 in different quality categories. Statistical significance was tested for all samples with the Kruskal-Wallis H-test for independent samples ($p = 0.2915$). (**b**) Long term stability of Glymo-functionalized glass, measured by the amount of dendrites growing outside of cell patterns at DIV 5. Statistical significance was tested for all samples with the Kruskal-Wallis H-test for independent samples ($p = 0.0037$). For individual comparison, Dunn's multiple comparison test with Bonferroni correction was used (p values < 0.05: $p_{week0/week4} = 0.0288$; see Supplementary Table S2)

Table S1. Results of Dunn's test with Bonferroni corrected p values for cells outside of patterns established with Glymo and DDA background hydrophobization (compare Figure 6).

Comparison	n1	Median 1	n2	Median 2	Corrected p Value
Week 0 vs. Week 4	12	5.5	9	3	1
Week 0 vs. Week 6	12	5.5	9	1	1
Week 0 vs. Week 7	12	5.5	9	9	1
Week 0 vs. Week 8	12	5.5	8	7.5	1
Week 0 vs. Week 10	12	5.5	6	1	0.395684
Week 0 vs. Week 22	12	5.5	6	2.5	1
Week 4 vs. Week 6	9	3	9	1	1
Week 4 vs. Week 7	9	3	9	9	0.219387
Week 4 vs. Week 8	9	3	8	7.5	0.782381
Week 4 vs. Week 10	9	3	6	1	1
Week 4 vs. Week 22	9	3	6	2.5	1
Week 6 vs. Week 7	9	1	9	9	0.0708654
Week 6 vs. Week 8	9	1	8	7.5	0.306228
Week 6 vs. Week 10	9	1	6	1	1
Week 6 vs. Week 22	9	1	6	2.5	1
Week 7 vs. Week 8	9	9	8	7.5	1
Week 7 vs. Week 10	9	9	6	1	0.00439755
Week 7 vs. Week 22	9	9	6	2.5	0.222034
Week 8 vs. Week 10	8	7.5	6	1	0.0236697
Week 8 vs. Week 22	8	7.5	6	2.5	0.690206
Week 10 vs. Week 22	6	1	6	2.5	1

Table S2. Results of Dunn's test with Bonferroni corrected p values for neurites outside of patterns established with Glymo and DDA background hydrophobization (compare Supplementary Figure S4b).

Comparison	n1	Median 1	n2	Median 2	Corrected p Value
Week 0 vs. Week 4	12	3.5	9	0	0.0288304
Week 0 vs. Week 6	12	3.5	9	3	1
Week 0 vs. Week 7	12	3.5	9	0	0.560302
Week 0 vs. Week 8	12	3.5	8	0.5	1
Week 0 vs. Week 10	12	3.5	6	1	1
Week 0 vs. Week 22	12	3.5	6	0	0.156157
Week 4 vs. Week 6	9	0	9	3	0.142673
Week 4 vs. Week 7	9	0	9	0	1
Week 4 vs. Week 8	9	0	8	0.5	1
Week 4 vs. Week 10	9	0	6	1	1
Week 4 vs. Week 22	9	0	6	0	1
Week 6 vs. Week 7	9	3	9	0	1
Week 6 vs. Week 8	9	3	8	0.5	1
Week 6 vs. Week 10	9	3	6	1	1
Week 6 vs. Week 22	9	3	6	0	0.473364
Week 7 vs. Week 8	9	0	8	0.5	1
Week 7 vs. Week 10	9	0	6	1	1
Week 7 vs. Week 22	9	0	6	0	1
Week 8 vs. Week 10	8	0.5	6	1	1
Week 8 vs. Week 22	8	0.5	6	0	1
Week 10 vs. Week 22	6	1	6	0	1
